# A nurse-led standardized bedside gastric ultrasound monitoring protocol for abdominal distension management in critically ill patients receiving enteral nutrition: a pragmatic, exploratory randomized controlled trial

**DOI:** 10.3389/fmed.2026.1902932

**Published:** 2026-10-14

**Authors:** Panpan Han, Ruxin Jiang, Huiyi Zhang

**Affiliations:** Department of Anesthesiology, Henan Provincial People’s Hospital, People’s Hospital of Zhengzhou University, Zhengzhou, China

**Keywords:** abdominal distension, bedside gastric ultrasound, critically ill patients, enteral nutrition, gastric residual volume, intra-abdominal pressure, nurse-led, standardized protocol

## Abstract

**Objective:**

To evaluate the feasibility and exploratory clinical effects of a standardized, nurse-led bedside gastric ultrasound-guided monitoring protocol for abdominal distension management in critically ill patients receiving enteral nutrition.

**Methods:**

In this single-center, pragmatic, exploratory randomized controlled trial, critically ill adults receiving enteral nutrition in the intensive care unit of Henan Provincial People's Hospital were assigned 1:1 to ultrasound-guided gastric residual volume (GRV) monitoring or syringe-aspiration monitoring. The primary outcome was abdominal circumference on day 7. Secondary outcomes included intra-abdominal pressure (IAP), exploratory biomarkers, gastric retention, feeding intolerance, and implementation measures.

**Results:**

Of 112 patients assessed, 100 were randomized and 91 completed the 7-day protocol, giving an overall retention rate of 91%. In the intervention group, the median number of ultrasound assessments was 14 per patient (IQR, 10–19), and protocol adherence was 82/89 (92.1%). Day-7 abdominal circumference was lower in the intervention group after baseline adjustment (adjusted mean difference, −2.35 cm; 95% CI, −3.28 to −1.43; *P* < 0.001). Baseline-adjusted IAP was also lower (−1.68 mmHg; 95% CI, −2.51 to −0.86; *P* < 0.001). Feeding intolerance did not differ significantly between groups (31.9% vs. 47.7%; RR, 0.67; 95% CI, 0.40–1.12; Fisher exact *P* = 0.139). Albumin was higher after baseline adjustment, whereas prealbumin did not differ significantly.

**Conclusion:**

The nurse-led protocol was feasible in this single-center population of non-intubated, relatively hemodynamically stable ICU patients and provided preliminary estimates for abdominal distension-related outcomes. The albumin result was exploratory and should not be taken as evidence of improved nutritional delivery. Secondary analyses were not adjusted for multiplicity and require cautious interpretation. The lack of prospective registration is a major limitation, and the findings do not apply to mechanically ventilated or hemodynamically unstable patients.

**Trial registration:**

The randomized design, outcomes, and analysis plan were specified in the ethics-approved protocol before enrollment. However, the separate requirement for registration in a public trial registry was overlooked, and the study was not prospectively registered [Ethics Committee of Henan Provincial People's Hospital, approval No. 2023(Ethics)127].

## 1 Introduction

Enteral nutrition (EN) is the preferred route of nutritional support for critically ill patients (1, 2). Early EN helps preserve the gastrointestinal barrier and intestinal function and may improve clinical outcomes (3, 4). However, disease severity, prolonged bed rest, sedatives, vasoactive drugs, and impaired gastrointestinal motility commonly cause gastrointestinal dysfunction in this population (5, 6). Abdominal distension during EN may interrupt feeding, reduce energy delivery, increase intra-abdominal pressure (IAP), impair gastrointestinal perfusion, and worsen nutritional status (7). Its timely recognition and management are therefore important to effective nutritional support in the intensive care unit (ICU).

Gastric residual volume (GRV) monitoring is commonly used to identify and manage EN-related abdominal distension (1, 8). Conventional syringe aspiration remains widespread but is inconvenient, may displace the nasogastric tube or introduce contamination, and provides an imprecise estimate of gastric contents (9). Bedside gastric ultrasound offers a non-invasive, real-time, repeatable alternative based on the gastric antral cross-sectional area. Its suitability for repeated bedside assessment makes it particularly relevant to nurse-led monitoring (10, 11). A standardized nursing protocol could link consistent GRV assessment to timely, protocol-based adjustments in feeding care (12).

Existing evidence has mainly established the technical feasibility and measurement validity of gastric ultrasound (13). A prospective multicenter cohort found that gastric suctioning was insufficiently reliable for assessing residual gastric contents because many patients retained a full stomach after aspiration (14). Trained ICU nurses can also perform repeated ultrasound assessments of GRV and nasogastric tube position (11). Observational evidence links IAP with EN tolerance; Bejarano et al. found that baseline IAP, together with APACHE II score, predicted feeding tolerance (15). However, these studies evaluated measurement accuracy or feasibility rather than whether an ultrasound-informed nursing protocol improves abdominal distension or IAP (11, 13, 14, 16). Randomized evidence linking nurse-led, protocol-based ultrasound monitoring to patient outcomes therefore remains limited.

We conducted a pragmatic, exploratory randomized controlled trial to assess the feasibility of a standardized, nurse-led bedside gastric ultrasound-guided protocol and to estimate its clinical effects on abdominal distension management in critically ill patients receiving EN. We hypothesized that the protocol would be associated with smaller abdominal circumference and lower IAP than conventional syringe-aspiration monitoring. GRV-related process measures and nutritional biomarkers were exploratory outcomes intended to inform the design of a future adequately powered trial.

## 2 Methods

### 2.1 Study design and participants

This single-center randomized controlled trial was conducted in the Anesthesia Intensive Care Unit (AICU) of Henan Provincial People's Hospital from January 2024 to March 2025. The Ethics Committee of Henan Provincial People's Hospital approved the study [approval No. 2023(Ethics)127]. Patients or their legal representatives provided written informed consent. The randomized design, outcomes, and analysis plan were specified in the ethics-approved protocol before enrollment. The team nevertheless overlooked the separate requirement for registration in a public trial registry, and the trial was not prospectively registered. This omission limits transparency but does not reflect a post-enrollment change in study design or outcomes.

The inclusion criteria were (1) age ≥18 years, (2) receipt of EN support, and (3) an expected ICU stay of ≥7 days. The exclusion criteria were (1) inability to visualize the gastric antrum because of substantial subcutaneous emphysema, an open abdominal wound, massive ascites, or previous gastric surgery; (2) pregnancy or acute pancreatitis; (3) known or suspected gastric outlet or bowel obstruction; (4) hemodynamic instability requiring high-dose vasopressors (norepinephrine >0.3 μg/kg/min or equivalent) at enrollment; (5) an expected survival of <48 h, as assessed by the attending physician; (6) invasive mechanical ventilation at enrollment; or (7) refusal to participate.

### 2.2 Randomization and allocation concealment

A random-number table was used to generate the allocation sequence, and participants were assigned 1:1 to the intervention or control group. An independent staff member kept the assignments in sequentially numbered, opaque, sealed envelopes, which were opened after enrollment. The visibly different ultrasound and syringe-aspiration procedures precluded blinding of nurses, patients, and bedside outcome assessors. Laboratory outcomes (ALB, PA, Hb, and lymphocyte count) were obtained from automated hospital systems by personnel uninvolved in the intervention, and the analyst was blinded to allocation. Standardized procedures for abdominal circumference and IAP measurement reduced, but could not eliminate, detection bias.

### 2.3 Sample size calculation

The sample size was based on abdominal circumference, the primary outcome. We assumed a between-group difference of 3.5 cm and a pooled standard deviation of 6 cm based on previous reports of abdominal monitoring in critically ill patients. With a two-sided alpha of 0.05 and 80% power, 45 participants were required per group. Allowing for 10% attrition, the planned sample was 50 participants per group, or 100 in total. Secondary outcomes were IAP, ALB, PA, hemoglobin (Hb), lymphocyte count, and feeding intolerance.

### 2.4 Interventions

#### 2.4.1 Research team and training

The research team was established in January 2024 and included one ICU head nurse, four ICU nurses, two clinicians, and one ultrasonographer. Two experienced ICU nurses performed all gastric ultrasound assessments. Before recruitment, both completed a standardized two-week program comprising 4 h of theoretical instruction and 12 h of hands-on practice, five expert demonstrations, at least 15 supervised scans, and 20 independent examinations. Qualification required a theoretical score of at least 90% and an acceptable image-acquisition rate of at least 90% in 10 double-blinded test subjects, with clear visualization of the gastric antrum and relevant anatomical landmarks. Only nurses who met all prespecified criteria performed study scans. During the study, the team randomly audited 10% of saved images each month to identify protocol drift. These procedures standardized image acquisition and provided continuing quality control during the trial.

#### 2.4.2 Intervention group

Patients assigned to the intervention group received standardized, nurse-led bedside gastric ultrasound monitoring during the 7-day study period. EN was delivered mainly by continuous pump infusion and was not paused for ultrasound assessment. Examinations were scheduled twice daily at 08:00 and 20:00 and were repeated when abdominal distension, vomiting or regurgitation, suspected aspiration, or clinical concern about feeding tolerance occurred. The gastric antrum was identified in a sagittal or oblique sagittal plane between the left lobe of the liver and the pancreas or aorta. In accordance with the Perlas model, antral cross-sectional area (CSA) was measured in the right lateral decubitus position at end-expiration. When automated free tracing was unavailable, CSA was calculated from the anteroposterior and craniocaudal diameters using CSA = (AP  ×  CC × π)/4. Each diameter was measured three times, and the mean was used. GRV was estimated as 27.0 + 14.6 × CSA (cm^2^) − 1.28 × age (years) (17). Because this equation was developed outside a continuously fed ICU population, the estimate was used to guide the prespecified protocol and was not treated as an exact physiological volume.

When estimated GRV exceeded 200 mL on two consecutive assessments or 300 mL on one assessment, the prespecified algorithm was activated. These values were safety-alert thresholds for reassessment and stepwise management, not substitutes for the ESPEN threshold of >500 mL/6 h for delaying EN and not mandates for prolonged feeding cessation (1). The repeated 200-mL criterion was selected because an adjusted observational study in surgical ICU patients linked repeated GRV >200 mL with aspiration, and similar lower thresholds have been studied in neurocritical care (18, 19). The same thresholds were used in both randomized groups. Protocol actions comprised head-of-bed elevation to at least 30°, a 50% reduction in the EN rate or a temporary 1-h interruption followed by reassessment, physician notification, prescribed prokinetic therapy when elevation persisted, and standardized abdominal massage when appropriate. The prescribed rate was restored after two consecutive values below the threshold. EN was given through a nasogastric tube using a polymeric formula, with target energy of 25–30 kcal/kg/day and protein of 1.2–1.5 g/kg/day. Actual delivered energy, protein, duration of rate reduction, and cumulative interruption time were not recorded; the effect of these actions on target attainment could therefore not be determined.

#### 2.4.3 Control group

The control group underwent standardized syringe-aspiration GRV monitoring every 4 h (six assessments per day) and received the same symptom-triggered additional assessments as the intervention group. The same operational thresholds, GRV >200 mL on two consecutive assessments or >300 mL once, activated the same supportive management algorithm. Under the control-group SOP, pump feeding was paused only for aspiration, which was completed within 5 min. Nurses aspirated the gastric contents, recorded the volume, slowly returned the aspirate, flushed the tube, and immediately resumed the prescribed infusion rate if the threshold was not reached. Unjustified extension of the interruption was prohibited. The brief procedure-related interruptions were intrinsic to syringe aspiration. Their cumulative effect on nutritional delivery could not be quantified because actual energy and protein delivery were not recorded. Nurse staffing and bedside observation were similar across groups, with a nurse-to-patient ratio of 1:3; however, the frequency and content of the two monitoring procedures were not formally time-matched.

### 2.5 Outcomes

The primary outcome was abdominal distension, operationalized as abdominal circumference on day 7. A designated nurse measured circumference at 08:00 with the patient supine and a soft tape positioned horizontally at the umbilicus. The same nurse performed all measurements to limit inter-rater variation, but group assignment could not be concealed because the monitoring procedures were visibly different. Formal test-retest reliability was not assessed.

Secondary outcomes included IAP, ALB, PA, Hb, lymphocyte count, and feeding intolerance. IAP was measured daily at 08:00 by transbladder manometry following World Society of Abdominal Compartment Syndrome guidance (20). With the patient supine, 25 mL of sterile saline at 37 °C was instilled through the urinary catheter. Pressure was recorded at end-expiration after zeroing the transducer at the mid-axillary line at the iliac crest; the day-7 value was analyzed. Feeding intolerance was defined as at least one episode of aspiration, protocol-defined gastric retention, or diarrhea during follow-up (21). Aspiration required gastric contents in the oropharynx or trachea. Gastric retention required at least one high-GRV episode, defined as GRV >200 mL on two consecutive assessments or >300 mL once. Diarrhea was defined as ≥3 loose or liquid stools daily (Bristol Stool Form Scale types 5–7) (22) or stool output >200 g/day. Because delivered versus prescribed energy and protein and feeding-interruption duration were not recorded, ALB, PA, and lymphocyte count were treated as exploratory biomarkers rather than direct measures of nutritional delivery. APACHE II and SOFA scores characterized illness severity at ICU admission (23, 24). Low-dose vasopressor exposure was recorded as any vasoactive drug use within 24 h after enrollment among participants who did not meet the high-dose exclusion criterion. Neurological diagnosis comprised a primary ICU admission diagnosis of cerebrovascular disease, brain tumor surgery, traumatic brain injury, intracranial hemorrhage, ischemic stroke, or another central nervous system disorder.

### 2.6 Statistical analysis

Analyses were performed in R version 4.3.3. Normality was assessed with the Shapiro–Wilk test. Normally distributed continuous variables are reported as mean ± SD and were compared using independent-samples t tests. Non-normally distributed variables (IAP and ALB) are reported as median (IQR) and were compared using the Mann–Whitney U test. Categorical outcomes were assessed using Fisher's exact test. The primary analysis used the per-protocol population of 91 participants who completed the 7-day follow-up, with participants analyzed according to their randomized group. ANCOVA models adjusted each post-intervention continuous outcome for its corresponding baseline value (25). Binary outcomes are reported as RR with 95% CI; absolute risk reduction and number needed to treat were calculated for feeding intolerance. Rank-based ANCOVA and change-from-baseline analyses were used as robustness checks. No adjustment was made for multiple comparisons, and all secondary and exploratory analyses were interpreted as hypothesis-generating. A two-sided *P* < 0.05 was considered statistically significant. No ITT effect estimate was generated because day-7 outcomes were unavailable for the nine participants who did not complete follow-up; no unobserved outcomes were imputed or generated.

## 3 Results

### 3.1 Characteristics of the patients

Figure 1 shows participant flow. Of 112 patients screened, 100 were randomized equally between groups, and enrollment ceased after the prespecified sample size was reached. Baseline characteristics of all randomized participants are presented in Table 1. Nine participants did not complete the 7-day follow-up (intervention, *n* = 3; control, *n* = 6), resulting in a retention rate of 91%; group-specific reasons are provided in Figure 1. Multiple research team members blinded to group assignment reviewed each discontinuation and judged all nine to be unrelated to the assigned monitoring procedure, protocol-triggered intervention, or an intervention-related adverse event. Outcome analyses included 91 participants with observed day-7 measurements (47 intervention and 44 control).

**Figure 1:**
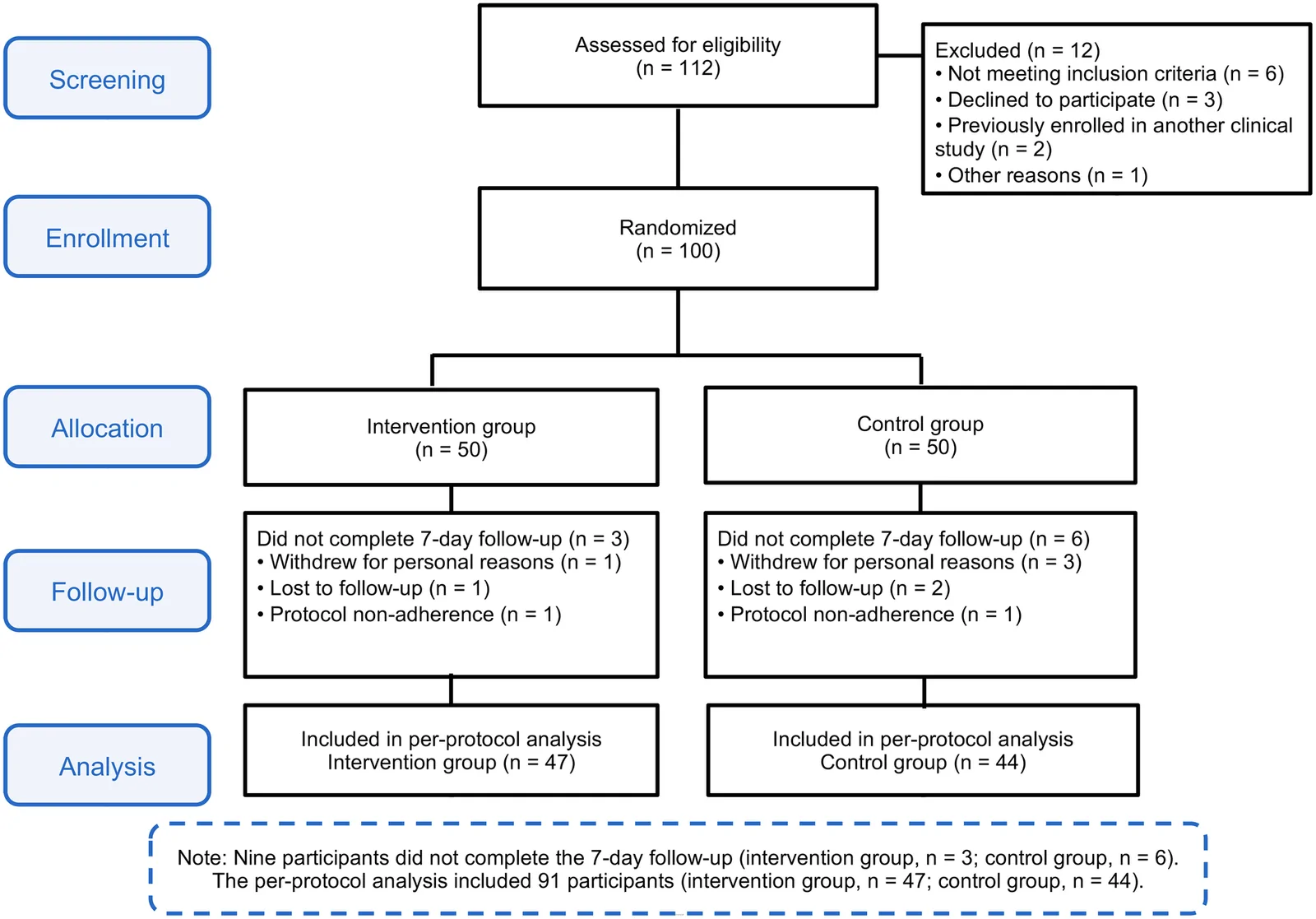
CONSORT flow diagram of participant screening, randomization, follow-up, and per-protocol analysis. Of 112 patients assessed for eligibility, 12 were excluded and 100 were randomized 1:1. Nine participants did not complete the 7-day follow-up. The per-protocol analysis included 91 participants, comprising 47 in the intervention group and 44 in the control group.

**Table 1:** Baseline characteristics of all randomized participants (*n* = 100).

Variable	Intervention group (*n* = 50)	Control group (*n* = 50)	*P*-value
Age (years)	60.44 ± 8.42	61.16 ± 8.11	0.664
BMI (kg/m^2^)	22.87 ± 0.80	23.09 ± 1.26	0.282
Male sex, *n* (%)	28 (56.0%)	29 (58.0%)	1.000
APACHE II score	22.0 ± 7.1	21.3 ± 6.0	0.597
SOFA score	8.7 ± 3.7	9.1 ± 3.6	0.642
Low-dose vasopressor use, *n* (%)	5 (10.0%)	7 (14.0%)	0.760
Neurological diagnosis, *n* (%)	27 (54.0%)	22 (44.0%)	0.424
Baseline abdominal circumference (cm)	84.46 ± 5.69	88.20 ± 5.01	0.001*
Baseline IAP (mmHg), median (IQR)	13.0 (11.2–16.8)	13.0 (11.2–16.0)	0.798
Baseline ALB (g/L)	35.87 ± 8.25	30.87 ± 6.59	0.001*
Baseline PA (mg/L)	116.17 ± 34.72	131.93 ± 43.96	0.050*
Baseline Hb (g/L)	104.92 ± 16.19	104.70 ± 15.27	0.944
Baseline lymphocyte count (×10⁹/L)	1.05 ± 0.25	1.19 ± 0.30	0.012*

Data are presented for all randomized participants (*n* = 100). IAP, intra-abdominal pressure; ALB, albumin; PA, prealbumin; Hb, hemoglobin; APACHE II, Acute Physiology and Chronic Health Evaluation II; SOFA, Sequential Organ Failure Assessment; ANCOVA, analysis of covariance. The baseline IAP *P* value was obtained using the Wilcoxon rank-sum test because of its non-normal distribution.

* *P* < 0.05. Baseline characteristics are descriptive for the randomized cohort. Outcome ANCOVA models used the corresponding baseline measure from the per-protocol population regardless of the baseline *P*-value.

### 3.2 Protocol implementation and GRV-guided nursing interventions

GRV-related process indicators were prospectively recorded in the intervention group to assess feasibility and implementation fidelity. Participants underwent a median of 14 ultrasound assessments (IQR, 10–19), and protocol adherence was 82/89 (92.1%). Eleven participants (23.4%) experienced at least one protocol-defined high-GRV episode, with 28 events in total. These 28 high-GRV events triggered 23 EN rate reductions and 18 temporary 1-h interruptions; actions were not mutually exclusive, and one event could prompt more than one response. They also triggered 15 prokinetic administrations and 26 abdominal-massage sessions. These counts describe implementation rather than comparative efficacy (Supplementary Table S1).

### 3.3 Primary outcome: abdominal circumference

Day-7 abdominal circumference was lower in the intervention group in the crude and baseline-adjusted analyses (adjusted mean difference, −2.35 cm; 95% CI, −3.28 to −1.43; *P* < 0.001; Table 2). The adjusted difference was smaller than the 3.5-cm difference assumed for sample-size planning. Change from baseline was directionally consistent (−3.34 vs. −1.00 cm).

**Table 2:** Abdominal circumference and intra-abdominal pressure.

Outcome	Time/analysis	Intervention group (*n* = 47)	Control group (*n* = 44)	Difference (95% CI)	Statistic	*P*-value
Abdominal circumference (cm)	Baseline	84.57 ± 5.84	87.97 ± 5.20	—	—	0.004
Abdominal circumference (cm)	Day 7	81.23 ± 6.07	86.97 ± 5.77	−5.74 (−8.21 to −3.28)	t = −4.629	<0.001
Abdominal circumference (cm)	ANCOVA adjusted	82.87	85.22	−2.35 (−3.28 to −1.43)	F = 25.497	<0.001
IAP (mmHg)	Baseline	13.0 (12.0–17.0)	13.0 (11.0–16.0)	—	W = 1119.5	0.498
IAP (mmHg)	Day 7	11.0 (9–13)	12.0 (9–15)	−1.0 (−3.0 to 1.0)	W = 880	0.223
IAP (mmHg)	ANCOVA adjusted	11.55	13.23	−1.68 (−2.51 to −0.86)	F = 16.532	<0.001

IAP was non-normally distributed and is presented as median (IQR) in crude analyses. ANCOVA estimates are adjusted marginal means. The abdominal-circumference model adjusted for baseline abdominal circumference, and the IAP model adjusted for baseline IAP. Baseline values refer to the per-protocol population.

### 3.4 Secondary outcomes

Gastric retention, defined as at least one protocol-defined high-GRV episode, occurred in 11 intervention participants (23.4%) and 17 controls (38.6%) (RR, 0.61; 95% CI, 0.32–1.15; *P* = 0.172; Table 3). GRV was estimated by ultrasound during ongoing EN in the intervention group and measured by syringe aspiration after a brief pump pause in controls. These measures were treated as protocol-related process indicators rather than interchangeable physiological measurements. Feeding intolerance and its individual components did not differ significantly between groups (Table 3). The study was not powered for these categorical outcomes.

**Table 3:** GRV-related events, feeding intolerance, and gastrointestinal complications during the 7-day intervention period.

Outcome		Intervention group (*n* = 47)	Control group (*n* = 44)	*P*-value	RR (95% CI)
GRV-related process indicator					
	Gastric retention (defined as ≥1 high-GRV episode), *n* (%)	11 (23.4%)	17 (38.6%)	0.172	0.61 (0.32 to 1.15)
Feeding intolerance and related complications					
	Feeding intolerance (composite), *n* (%)	15 (31.9%)	21 (47.7%)	0.139	0.67 (0.40 to 1.12)
	Aspiration, *n* (%)	2 (4.3%)	3 (6.8%)	0.670	0.62 (0.11 to 3.56)
	Diarrhea, *n* (%)	6 (12.8%)	9 (20.5%)	0.402	0.62 (0.24 to 1.61)

RR, risk ratio; CI, confidence interval. *P* values were obtained using Fisher's exact test. Feeding intolerance was defined as aspiration, protocol-defined gastric retention, or diarrhea during the intervention period. Gastric retention was defined as at least one high-GRV episode: GRV >200 mL on two consecutive assessments or >300 mL on one assessment.

Crude post-intervention IAP did not differ between groups, whereas baseline-adjusted IAP was lower in the intervention group (adjusted mean difference, −1.68 mmHg; 95% CI, −2.51 to −0.86; *P* < 0.001; Table 2). Adjusted ALB was higher in the intervention group, whereas PA and Hb did not differ significantly. The adjusted lymphocyte-count result was exploratory because the crude difference was negligible (Table 4). No multiplicity correction was applied to secondary outcomes. The ANCOVA-adjusted between-group effects are summarized in Figure 2.

**Figure 2:**
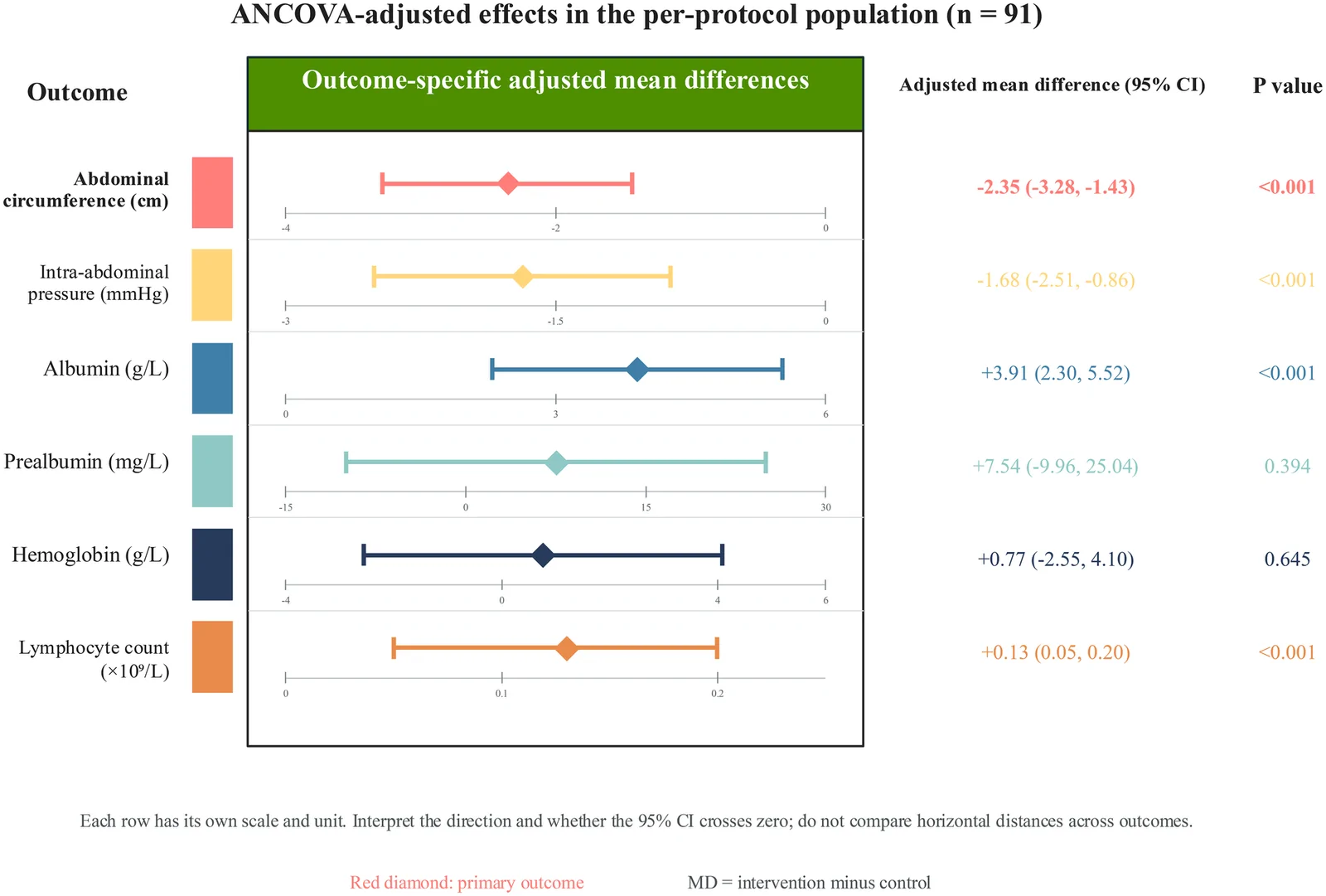
Forest plot of ANCOVA-adjusted between-group effects in the per-protocol population (*n* = 91). Markers show adjusted mean differences (intervention minus control), and horizontal bars show 95% CIs. Each model adjusted for the corresponding baseline value. The red diamond marks the primary outcome; each outcome is shown in a different color and on its own scale. Negative estimates for abdominal circumference and IAP and positive estimates for ALB and lymphocyte count favor the intervention group.

**Table 4:** Nutritional indicators after intervention.

Outcome	Intervention group (*n* = 47)	Control group (*n* = 44)	Crude difference (95% CI)	Statistic	*P*-value
ALB (g/L)	39.6 (34.3–48.7)	30.0 (25.9–35.5)	+9.6 (5.3 to 13.5)	W = 1605	<0.001
ALB, ANCOVA adjusted	37.51	33.60	+3.91 (2.30 to 5.52)	F = 23.340	<0.001
PA (mg/L)	142.28 ± 51.52	150.74 ± 55.65	−8.46 (−30.85 to 13.93)	t = −0.751	0.455
PA, ANCOVA adjusted	150.02	142.48	+7.54 (−9.96 to 25.04)	F = 0.733	0.394
Hb (g/L)	106.64 ± 18.00	106.23 ± 16.73	+0.41 (−6.83 to 7.64)	t = 0.112	0.911
Hb, ANCOVA adjusted	106.82	106.05	+0.77 (−2.55 to 4.10)	F = 0.213	0.645
Lymphocyte count (×10⁹/L)	1.19 ± 0.30	1.20 ± 0.34	−0.01 (−0.15 to 0.12)	t = −0.156	0.876
Lymphocyte count, ANCOVA adjusted	1.25	1.13	+0.13 (0.05 to 0.20)	F = 12.035	<0.001

ALB was non-normally distributed and is presented as median (IQR) in the crude analysis. ANCOVA estimates are adjusted marginal means. The ALB, PA, Hb, and lymphocyte-count models adjusted for their corresponding baseline values.

### 3.5 Analysis population and robustness checks

All reported outcome analyses used the per-protocol population of 91 participants. Rank-based ANCOVA and change-from-baseline analyses supported the direction of the adjusted abdominal-circumference, IAP, and ALB findings. The lymphocyte-count finding remained exploratory because the crude between-group difference was negligible. Nine randomized participants lacked day-7 outcome measurements and were excluded from outcome analyses; therefore, the study did not provide a valid ITT effect estimate.

## 4 Discussion

### 4.1 Protocol feasibility and implementation

The nurse-led protocol was feasible, with 91% retention, a median of 14 scans per intervention participant, and 92.1% adherence. Participants assigned to the protocol had smaller day-7 abdominal circumference and lower baseline-adjusted IAP. These results estimate the possible clinical effect of the management protocol; they do not show that ultrasound itself reduced gastric volume. Gastric-retention and feeding-intolerance events did not differ significantly, and the biomarker analyses were exploratory.

Ultrasound results were linked to prespecified bedside actions, including positioning, adjustment of the feeding rate, prescribed prokinetic treatment, and brief interruption when indicated. The trial therefore evaluated the complete management protocol rather than ultrasound measurement alone. Ultrasound was performed twice daily without stopping EN, whereas aspiration was performed every 4 h and required a short pump pause. Both groups had the same symptom triggers, operational thresholds, management algorithm, staffing ratio, and routine bedside observation. The procedures were not time-matched, however, and their intrinsic differences prevent the effect of ultrasound information from being separated fully from measurement and co-intervention effects.

### 4.2 Lower intra-abdominal pressure: a consistent but mechanistically unproven signal

Baseline-adjusted IAP was lower in the intervention group. We do not interpret this finding as evidence of improved splanchnic perfusion or restored gastrointestinal motility because the study measured pressure but not mucosal perfusion, gastric emptying, or regional blood flow. The data support the narrower conclusion that an ultrasound-informed management bundle accompanied lower IAP, while the physiological intermediates remain uncertain.

The direction of this finding agrees with reports in which IAP-guided feeding adjustment improved tolerance or reduced feeding-related complications; Bejarano et al. also identified IAP as a prognostic marker of EN tolerance (15). By contrast, routine GRV monitoring did not improve outcomes in a randomized trial of mechanically ventilated patients (26). Although that population differed from ours, the comparison suggests that measurement alone may be insufficient without an action-oriented response. Differences in monitoring thresholds, response algorithms, populations, and endpoints may also explain the discordance. Our study measured a graded physiological signal within an active protocol rather than a downstream clinical event under passive monitoring. IAP should therefore be viewed as one component of the management pathway, not as a stand-alone target. Future trials should test whether changes of this magnitude mediate clinical benefit by measuring IAP alongside motility or perfusion surrogates and patient-centered endpoints.

### 4.3 Interpretation of the albumin finding

ALB was higher in the ultrasound-guided group after baseline adjustment, but the study did not measure the variables needed to attribute this difference to nutritional delivery. Delivered energy and protein, cumulative feeding-interruption duration, inflammatory markers, and fluid balance were not recorded, and baseline ALB differed between groups. Albumin is a negative acute-phase reactant affected by inflammation, capillary leakage, fluid shifts, hepatic synthesis, renal loss, and disease severity (27). PA, which is more responsive to short-term change, did not differ significantly. This discordance further weakens a nutritional interpretation of the ALB result. The ALB finding should therefore be treated as exploratory, especially because secondary outcomes were not adjusted for multiplicity. Future studies should measure nutrition delivery, target attainment, inflammation, and fluid balance directly.

### 4.4 Feeding intolerance: exploratory and underpowered categorical outcomes

Feeding intolerance did not differ significantly between groups. The trial was powered for abdominal circumference and was underpowered for infrequent categorical outcomes, including aspiration. Accordingly, the point estimates cannot support claims of prevention or a favorable trend. A larger trial should prespecify an event-based primary outcome and use these estimates only for sample-size planning (28).

This distinction also clarifies the relationship between our approach and controlled enteral-delivery systems (29). Such systems narrow the gap between prescribed and delivered nutrition, whereas our protocol uses dynamic monitoring to support feeding tolerance. The approaches are complementary. However, whether improved tolerance facilitates nutritional delivery cannot be determined without direct measurements of delivered energy, protein, and feeding interruptions. Future trials should evaluate both strategies and be powered for feeding-intolerance events.

### 4.5 Clinical implications

The protocol was delivered by two trained ICU nurses using defined imaging, measurement, documentation, and decision steps. Monthly image audits were used to monitor adherence to the scanning procedure. The implementation results show that the protocol could be incorporated into bedside care and that ultrasound findings informed predefined nursing actions. Previous observational studies have also supported the feasibility of ultrasound assessment of gastric volume in critically ill patients (30, 31). They do not establish that ultrasound-derived GRV is superior to aspiration-derived GRV or that the protocol improved nutritional delivery. Any future multicenter trial evaluating this protocol should be prospectively registered in a publicly accessible trial registry before enrollment of the first participant. It should also include time-matched process measures, direct measures of delivered energy and protein, and formal assessment of measurement reliability.

### 4.6 Limitations

Several considerations should guide the interpretation of these findings. This was a single-center study that was not prospectively registered. Future confirmatory multicenter trials should be registered in a publicly accessible registry before enrollment of the first participant. The study population was restricted to non-intubated, relatively hemodynamically stable ICU patients, which limits generalizability to mechanically ventilated patients or those requiring substantial vasopressor support. Abdominal circumference provided a practical bedside measure of distension but is not specific to gastric volume and may also be influenced by bowel gas, edema, ascites, positioning, and measurement variability. Because the monitoring procedures were visibly different, blinding was not feasible, and differences in monitoring frequency and brief aspiration-related feeding pauses may have contributed to the observed effects. Ultrasound-estimated and aspiration-measured GRV should also be regarded as protocol-specific measures rather than directly interchangeable physiological measurements. Finally, actual energy and protein delivery, cumulative feeding interruption, inflammatory markers, and fluid balance were not recorded; therefore, nutritional biomarkers and other secondary outcomes remain exploratory. Day-7 outcomes were analyzed in 91 completers, with unequal attrition potentially biasing the per-protocol estimates. Moreover, the study was not powered to detect differences in categorical complications, including feeding intolerance and aspiration.

## 5 Conclusion

In non-intubated, relatively hemodynamically stable ICU patients receiving EN, the nurse-led ultrasound-guided protocol was feasible and yielded preliminary estimates for abdominal distension-related outcomes. The study did not determine whether ultrasound reduced gastric volume or improved nutritional delivery. The baseline-adjusted between-group difference of 2.35 cm was smaller than the 3.5-cm difference assumed during sample-size planning. The observed estimate, together with its confidence interval and an allowance for attrition, should therefore inform the sample-size calculation of a future confirmatory trial. Confirmation requires a prospectively registered, adequately powered multicenter trial with ICU-appropriate gastric measurements, direct nutrition-delivery outcomes, formal reliability assessment, and time-matched process evaluation.

## Data Availability

The raw data supporting the conclusions of this article will be made available by the authors, without undue reservation.

## Supplementary material

Supplementary Table S1 Protocol-triggered nursing interventions based on GRV assessment in the ultrasound-guided group during the 7-day intervention period.
