# Supplementary material for: A nurse-led standardized bedside gastric ultrasound monitoring protocol for abdominal distension management in critically ill patients receiving enteral nutrition: a pragmatic, exploratory randomized controlled trial
Source: Front Med (Lausanne). 2026 Oct 14;13:1902932. doi: 10.3389/fmed.2026.1902932 (PMC13713614; doi:10.3389/fmed.2026.1902932)
Supplement: Supplementary file 1 [file Datasheet1.pdf]

CONSORT 2010 Checklist

Manuscript title: A Nurse-Led Standardized Bedside Gastric Ultrasound Monitoring Protocol for Abdominal Distension Management in Critically Ill Patients Receiving Enteral Nutrition: A Pragmatic Pilot Randomized Controlled Trial

ID: 1902932

Journal/section: Frontiers in Medicine — Intensive Care Medicine and Anesthesiology

Note: Page and line locations refer to the V2 revised manuscript with continuous line numbering. The trial was not prospectively registered, and a publicly accessible full protocol is not reported.

Section/topic	Item	Checklist item	Reported on page/line
**Title and abstract**	**1a**	Identification as a randomized trial in the title.	p. 1, title
**Title and abstract**	**1b**	Structured summary of trial design, methods, results, and conclusions.	p. 1–2, lines 9–39
**Background and objectives**	**2a**	Scientific background and explanation of rationale.	p. 2, lines 42–66
**Background and objectives**	**2b**	Specific objectives or hypotheses.	p. 2, lines 67–72
**Trial design**	**3a**	Description of trial design, including allocation ratio.	p. 3, lines 75–90
**Trial design**	**3b**	Important changes to methods after trial commencement, with reasons.	no changes are described
**Participants**	**4a**	Eligibility criteria for participants.	p. 3, lines 80–86
**Participants**	**4b**	Settings and locations where data were collected.	p. 3, lines 75–79
**Interventions**	**5**	Interventions for each group with sufficient detail to allow replication.	pp. 3–4, lines 103–148
**Outcomes**	**6a**	Completely defined prespecified primary and secondary outcomes, including how and when assessed.	pp. 4–5, lines 149–172
**Outcomes**	**6b**	Changes to trial outcomes after commencement, with reasons.	no outcome changes are described
**Sample size**	**7a**	How sample size was determined.	p. 3, lines 96–102
**Sample size**	**7b**	Explanation of interim analyses and stopping guidelines, when applicable.	N/A — no interim analysis or early stopping rule
**Randomisation: sequence generation**	**8a**	Method used to generate the random allocation sequence.	p. 3, lines 88–89
**Randomisation: sequence generation**	**8b**	Type of randomisation and details of any restriction.	p. 3, lines 88–90
**Allocation concealment**	**9**	Mechanism used to implement the random allocation sequence and steps taken to conceal it.	p. 3, lines 89–90
**Implementation**	**10**	Who generated the sequence, enrolled participants, and assigned interventions.	p. 3, lines 88–90; independent staff member managed envelopes
**Blinding**	**11a**	Who was blinded after assignment and how.	p. 3, lines 91–95
**Blinding**	**11b**	If relevant, description of similarity of interventions.	p. 3, lines 91–95; procedural blinding was not feasible
**Statistical methods**	**12a**	Methods used to compare groups for primary and secondary outcomes.	p. 5, lines 173–190
**Statistical methods**	**12b**	Methods for additional analyses, such as subgroup and adjusted analyses.	p. 5, lines 179–190; p. 7, lines 229–234
**Participant flow**	**13a**	Numbers randomly assigned, receiving intended treatment, and analysed for primary outcome.	pp. 5–6, lines 193–202; Figure 1
**Participant flow**	**13b**	Losses and exclusions after randomisation, with reasons.	p. 5, lines 194–197; Figure 1
**Recruitment**	**14a**	Dates defining periods of recruitment and follow-up.	p. 3, lines 75–76
**Recruitment**	**14b**	Why the trial ended or was stopped.	p. 5, lines 193–194
**Baseline data**	**15**	Table showing baseline demographic and clinical characteristics for each group.	p. 13, lines 460–467; Table 1
**Numbers analysed**	**16**	For each group, denominator included in each analysis and whether analysed by original assignment.	p. 5, lines 193–198; Tables 2–4, p. 14–15
**Outcomes and estimation**	**17a**	Results for each primary and secondary outcome, effect size, and precision.	pp. 6–7, lines 211–227; Tables 2–4
**Outcomes and estimation**	**17b**	For binary outcomes, both absolute and relative effect sizes.	p. 1, lines 27–30; p. 7, lines 225–227; Table 4
**Ancillary analyses**	**18**	Results of other analyses, distinguishing prespecified from exploratory.	p. 5, lines 179–190; p. 7, lines 229–234
**Harms**	**19**	All important harms or unintended effects in each group.	p. 1, lines 30–31
**Limitations**	**20**	Trial limitations, including bias, imprecision, and multiplicity.	p. 9, lines 313–330
**Generalisability**	**21**	Generalisability (external validity, applicability) of findings.	p. 9, lines 328–330
**Interpretation**	**22**	Interpretation consistent with results, balancing benefits and harms and considering other evidence.	p. 7–10, lines 239–339
**Registration**	**23**	Registration number and name of trial registry.	p. 2, lines 38–39; p. 9, lines 327–328 — trial not prospectively registered
**Protocol**	**24**	Where the full trial protocol can be accessed, if available.	full protocol not publicly available
**Funding**	**25**	Sources of funding and other support and role of funders.	p. 10, lines 352–356
